# Genome-wide identification and characterization of the GRX gene family in *Castanea mollissima* under abiotic stresses

**DOI:** 10.3389/fpls.2026.1826022

**Published:** 2026-05-19

**Authors:** Xili Liu, Tong Zhang, Huoqiang Quan, Qinuo Li, Zhihui Wang, Dongsheng Wang, Guoyun Zhang, Haie Zhang, Xiangyu Wang, Liyang Yu

**Affiliations:** 1 Engineering Research Center of Chestnut Industry Technology, Ministry of Education, Hebei Normal University of Science and Technology, Qinhuangdao, Hebei, China; 2 Powerchina Northwest Engineering Corporation Limited, Xian, Shaanxi, China; 3 China Three Gorges University, College of Electrical Engineering and New Energy, Yichang, Hubei, China; 4 Research Institute of Forestry, Chinese Academy of Forestry Sciences, Beijing, China; 5 Hebei Key Laboratory of Horticultural Germplasm Excavation and Innovative Utilization, College of Horticulture Science and Technology, Hebei Normal University of Science and Technology, Qinhuangdao, Hebei, China; 6 The Office of Scientific Research, Hebei Normal University of Science and Technology, Qinhuangdao, Hebei, China

**Keywords:** abiotic stress, *Castanea mollissima*, expression profiling, GRX gene family, RT-qPCR

## Abstract

Glutaredoxins (GRXs) are versatile oxidoreductases that play a crucial role in maintaining cellular redox homeostasis and supporting plant development amid environmental changes. The Chinese chestnut (*Castanea mollissima*) is particularly notable for its dual value both as a significant woody food crop and as a pioneer species in soil and water conservation and ecological afforestation in the Northern Hemisphere. Despite its importance, the evolutionary characteristics of the *GRX* gene family in *C. mollissima*, as well as their mechanisms of response to abiotic stresses, are not well understood. In this study, we identified a total of 32 *CmGRX* genes within the *C. mollissima* genome, distributed across 12 chromosomes and one scaffold. Phylogenetic analysis categorized these genes into three subfamilies: CPYC, CGFS, and CC types. Evolutionary analysis suggests that dispersed duplication was the primary mechanism driving the expansion of the *CmGRX* gene family. Further investigations into *cis*-acting elements and transcriptional networks revealed that *CmGRX* genes are enriched with stress-responsive elements and are regulated by stress-related transcription factors, including ERF, MYB, and NAC. Moreover, transcriptomic data revealed distinct expression patterns of *CmGRX* members under various stresses such as high temperature, low temperature, salt-alkaline conditions, and shading, indicating their functional divergence in adaptation to these adversities. The reliability of the RNA-seq data was confirmed through RT-qPCR analysis. This study provides a comprehensive examination of the evolutionary history and stress-responsive expression regulatory network of the *CmGRX* gene family, offering valuable theoretical insights and gene resources for the development of resilient varieties aimed at ecological restoration and sustainable forestry in *C. mollissima*.

## Introduction

Reactive oxygen species (ROS), which include hydrogen peroxide (H_2_O_2_), singlet oxygen (¹O_2_), hydroxyl radicals (·OH), and superoxide radicals (O_2_^-^), are produced both during normal plant metabolism and under abiotic stress conditions. Owing to their high reactivity and toxicity, excessive ROS accumulation can damage proteins, lipids, carbohydrates, and DNA, ultimately leading to oxidative stress and impairing plant growth and development (Cruz De Carvalho, 2008; Gill and Tuteja, 2010; Swanson and Gilroy, 2010; Waszczak et al., 2018; Wang et al., 2024). Normally, ROS generation and scavenging are in dynamic equilibrium; however, this balance is disrupted when plants are exposed to biotic or abiotic stresses (Apel and Hirt, 2004; Foyer and Noctor, 2005; Gill and Tuteja, 2010; Mittler et al., 2022). Glutaredoxins (GRXs), a class of oxidoreductases found in animals, plants, and microorganisms, use glutathione (GSH) to reduce disulfide bonds in substrate proteins, thus protecting cells from oxidative damage and playing crucial roles in ROS signal transduction (Rouhier et al., 2004; Meyer et al., 2012; Chai and Mieyal, 2023). Based on the active site signature, GRXs are categorized into three types: CPYC (Class I), CGFS (Class II), and CC (Class III), with the latter being restricted to terrestrial plants. Specifically, Class III (CC-type) GRXs are unique to land plants (Holmgren, 1976; Rouhier et al., 2004; Mrozek et al., 2025). Since their initial discovery in *Escherichia coli* (Holmgren, 1976), *GRX* gene families have been identified in various plant species, including *Arabidopsis thaliana* (31) (Rouhier et al., 2004), *Oryza sativa* (48) (Garg et al., 2010), *Capsicum annuum* (35) (Guo et al., 2025), *Gossypium hirsutum* (127) (Malik et al., 2020), *Musa acuminata* (64) (Li et al., 2021), and *Puccinellia tenuiflora* (25) (Liu et al., 2025b).

Research has demonstrated that GRXs play a critical role in various biological processes, including plant growth and development, DNA synthesis, nitrogen fixation, stress responses, and signal transduction (Fernandes and Holmgren, 2004; Rouhier et al., 2004; Li and Outten, 2012; Alloing et al., 2018; Mondal et al., 2020; Jiménez et al., 2024). For example, the *A. thaliana* Class II member *GRXS15* is involved in the assembly of mitochondrial iron-sulfur (Fe-S) clusters. This activity affects the function of lipoic acid-dependent enzymes, thereby regulating plant growth and enhancing arsenic tolerance (Ströher et al., 2016). Another member, *AtGRXS8*, modulates nitrate responses by influencing the activity of the TGA1/TGA4 transcription factors (TFs) (Ehrary et al., 2020). The heterologous expression of *AtGRXS17* in tomatoes enhances their tolerance to drought and oxidative stress (Wu et al., 2017a). Similarly, the overexpression of the rice *OsGRX20* gene significantly improves resistance to bacterial leaf streak, methyl viologen, and salt stress (Ning et al., 2018). Furthermore, *A. thaliana* lines overexpressing two rice *GRX* genes (*LOC_Os02g40500* and *LOC_Os01g27140*) have shown improved root systems, higher seed germination rates, and increased survival rates under drought stress, accompanied by improved physiological and antioxidant indices (Kumar et al., 2020). In cassava, the gene *MeGRXC15* is induced by drought, and its overexpression enhances ABA tolerance in *A. thaliana*, influencing stress-related signaling pathways (Ruan et al., 2018). In *Puccinellia tenuiflora*, several *PutGRX* genes respond to saline-alkali stress, with *PutGrxS12* being critical for maintaining ROS homeostasis (Liu et al., 2025b). Despite extensive studies on the *GRX* gene family in numerous plant species, the membership and expression patterns under abiotic stresses in Chinese chestnut (*Castanea mollissima*) have not yet been reported.

*Castanea mollissima*, a deciduous tree belonging to the family Fagaceae, produces nuts that are rich in carbohydrates, proteins, and trace elements. *C. mollissima* is extensively cultivated in East Asia, where it holds significant economic importance (Borges et al., 2008; Massantini et al., 2021; Guo et al., 2022). Abiotic stresses, such as salinity, alkalinity, drought, extreme temperatures, and heavy metals, significantly limit crop yield (Nguyen et al., 2018). Salinity and drought stress induce osmotic pressure, which inhibits the growth of leaves and roots, thereby reducing yield and seed vigor after prolonged exposure (Arif et al., 2020; Shahzad et al., 2022). Alkaline stress leads to ion toxicity and oxidative damage, and directly disrupts the structure of chloroplasts, resulting in reduced photosynthetic activity (Soliman et al., 2011; 2012;Yang et al., 2024). Extreme temperatures impair plant metabolism, damage membrane systems, and inhibit photosynthesis (Ashraf and Harris, 2013). These stresses induce the excessive accumulation of ROS, severely affecting the growth, yield, and quality of *C. mollissima*. Consequently, the systematic identification of the *CmGRX* gene family and the elucidation of their functions under abiotic stresses are crucial for enhancing stress resistance and ensuring the sustainable development of the chestnut industry.

## Materials and methods

### Identification and phylogenetic analysis of *CmGRX* gene family members

The complete genome sequences of *C. mollissima* were obtained from the *Castanea* Genome Database (http://castaneadb.net/#/). To identify members of the *GRX* gene family, 31 GRX protein sequences from *A. thaliana* served as queries for BLASTP (v2.2.30) searches against the *C. mollissima* proteome with an E-value threshold of 1e-5. These candidates were subsequently filtered using the *GRX* family Hidden Markov Model (HMM) profile (PF00462) from the Pfam database through HMMER 3.0 software with an E-value cutoff below 1 × 10^-^³. Candidate proteins were verified for the presence of the typical GRX conserved domain using the NCBI-CDD (https://www.ncbi.nlm.nih.gov/cdd/) and SMART (http://smart.embl-heidelberg.de/) databases. Physicochemical properties were predicted using the ExPASy (https://web.expasy.org/protparam/) online tool, subcellular localization was predicted with WoLF PSORT (https://wolfpsort.hgc.jp/), and conserved motifs were identified using the MEME (https://meme-suite.org/meme/) suite. The gene structures were visualized using the genome annotation file (gff3) with TBtools (v2.363) (Chen et al., 2020). A Maximum Likelihood (ML) phylogenetic tree, incorporating GRX sequences from both *C. mollissima* and *A. thaliana*, was generated using the MEGA 11.0 package with the LG+I+G4 amino acid substitution model. Branch support was assessed using 5000 bootstrap replicates (Tamura et al., 2021).

### Chromosomal location and collinearity analysis

The physical locations of *CmGRX* genes on chromosomes were determined using genome annotation files and visualized with TBtools (Chen et al., 2020). To explore evolutionary relationships and gene duplication mechanisms, genome sequences for *A. thaliana*, *Quercus robur*, *Solanum lycopersicum*, *Vitis vinifera*, *Zea mays*, *O. sativa*, and *Pyrus bretschneideri* were obtained from the Phytozome (https://phytozome-next.jgi.doe.gov/) database (Goodstein et al., 2012; Liu et al., 2025a; Yu et al., 2025b). Synteny analysis within the *C. mollissima* genome was conducted using MCScanX. Specific duplication modes of *CmGRX* genes were categorized using the built-in “duplicate_gene_classifier” module (Wang et al., 2012). The differentiation between WGD and segmental duplication events was achieved by integrating data from self-collinearity dot plots, synonymous substitution rates (Ks) of syntenic pairs, and block complementarity, as previously described (Yu et al., 2022, 2025b).

### *Cis*-acting elements, transcriptional regulatory network, and functional enrichment analysis

*Cis*-acting elements within the 2000 bp promoter regions upstream of the start codon for each *GRX* gene were predicted using the PlantCARE (http://bioinformatics.psb.ugent.be/webtools/plantcare/html/) online tool (Lescot et al., 2002). Potential TF regulatory relationships were predicted using the binding site prediction function of the Plant Transcriptional Regulatory Map database (http://plantregmap.gao-lab.org/) (Tian et al., 2020). The 2000 bp promoter sequences upstream of the start codon for each *CmGRX* gene were submitted as input, with *A. thaliana* selected as the reference species. TF binding sites were identified using a significance threshold of P-value ≤ 1 × 10^-4^. The regulatory network was constructed and visualized using Cytoscape (v3.9.1) (Kohl et al., 2011).Furthermore, GO annotation and KEGG pathway enrichment analyzes for the *GRX* gene family were conducted and visualized using TBtools (Chen et al., 2020).

### Transcriptome analysis

The transcriptomic datasets for *C. mollissima* under various abiotic stresses were retrieved from public repositories. Data regarding temperature (high and low), salt, and alkaline stress were sourced from the NCBI SRA database, while shading stress data were obtained from the NGDC database (GSA: CRA022911). These resources are associated with the following accession numbers: PRJNA1166987 for temperature treatments (45 °C for 0, 4, 8, 12 h; -15 °C for 0, 5, 10, 15 h), PRJNA1363574 for salt stress (200 mmol/L NaCl for 14 and 26 days), and PRJNA1384844 for alkaline stress (0.02 and 0.5 g/L Na_2_CO_3_ for 7 days). For shading stress, the dataset encompassed four light intensities: 0% (SCK), 50%, 75%, and 90% shading. Gene expression levels were quantified using FPKM values, which were subsequently log2-transformed for normalization. Heatmaps were constructed using TBtools to visualize the expression patterns across these diverse environmental conditions.

### Plant materials and RT-qPCR validation

Healthy seedlings of *C. mollissima* ‘Yanshanzaofeng’, grown at theexperimental station of Hebei Normal University of Science and Technology, served as the primary experimental material. To evaluate gene responses to abiotic stress, seedlings were subjected to temperature extremes (-15 °C and 45 °C), salt stress (200 mmol/L NaCl), alkaline stress (0.02 g/L and 0.5 g/L Na_2_CO_3_), and varying shading levels (50%, 75%, and 90%). Following treatment, leaf tissues were immediately flash-frozen in liquid nitrogen and maintained at -80 °C. Total RNA was isolated and synthesized into first-strand cDNA using the Evo M-MLV RT Mix Kit with gDNA Clean. Real-time quantitative PCR (RT-qPCR) was performed on an ABI 7500 system using TB Green Premix Ex Taq (Takara). The thermal cycling conditions consisted of 95 °C for 3 min, followed by 40 cycles of 95 °C for 10 s and 60 °C for 20 s. The *C. mollissima* Actin gene was employed as an internal control, and gene-specific primers are listed in **Supplementary Table 1**. All experiments were conducted with three biological and three technical replicates. Relative expression levels were calculated using the 2^-△△CT^ method (Livak and Schmittgen, 2001).

## Results

### Identification, classification, and physicochemical characterization of *CmGRX* genes

**Figure 1:**
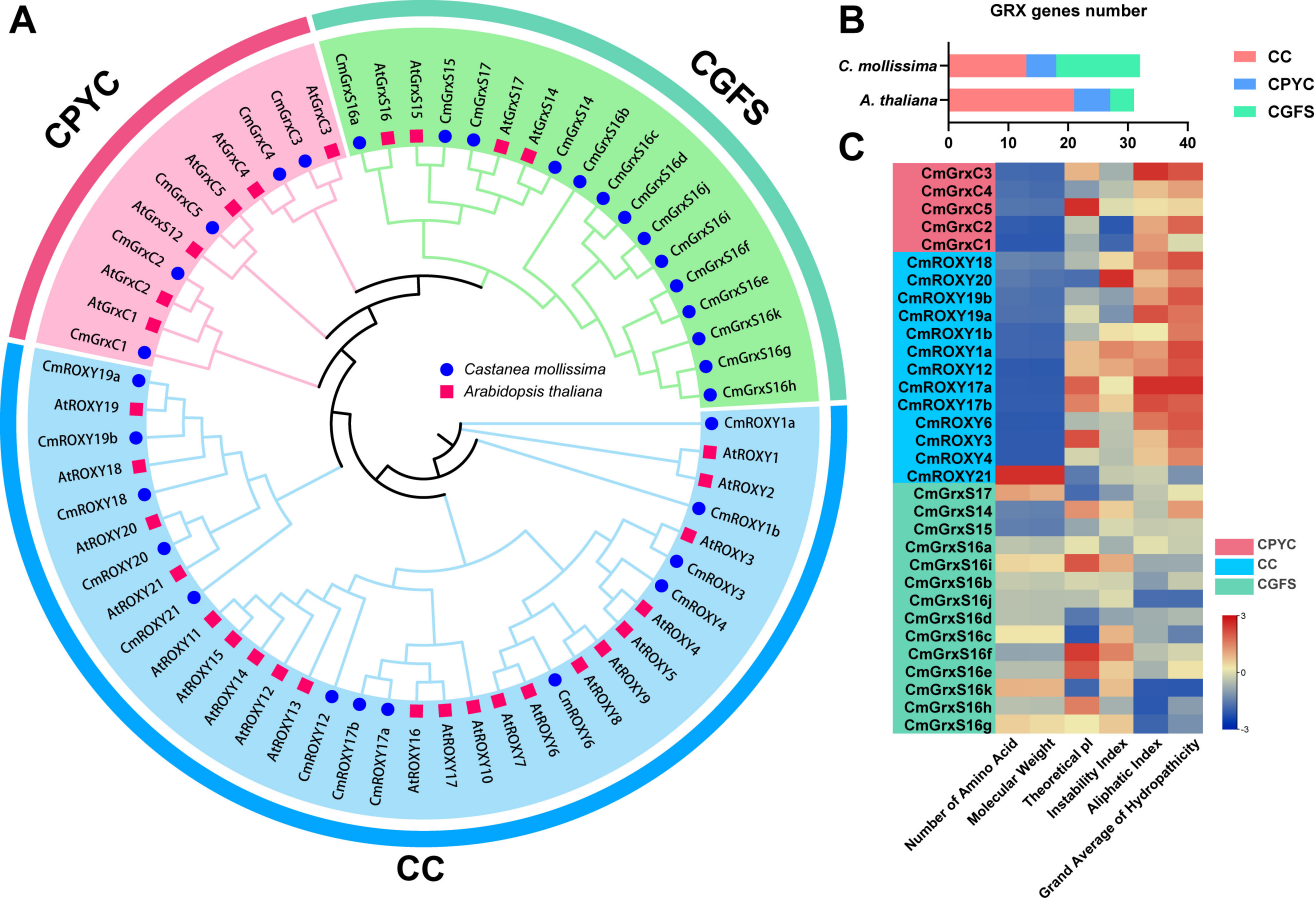
Phylogenetic analysis of the *GRX* genes of *C. mollissima* and *A. thaliana*. **(A)** Phylogenetic evolution of *GRX* members of *C. mollissima* (32) and *A. thaliana* (31). Different background colors represent different subfamilies. **(B)** Population distribution of *GRX* genes from *C. mollissima* and *A. thaliana*. **(C)** Heatmap of the physicochemical properties of GRX proteins.

A genome-wide identification in *C. mollissima* revealed a total of 32 *CmGRX* genes. These genes were named based on their phylogenetic relationships with homologs from *A. thaliana* and were classified into three subfamilies: the CPYC subfamily (5 members); the CGFS subfamily (14 members); and the land plant-specific CC subfamily (13 members) (**Supplementary Table 2**). Notably, a comparative analysis with *A. thaliana* indicated a higher proportion of CGFS members and a relatively lower proportion of CC members in *C. mollissima*, suggesting lineage-specific expansion and contraction of the *GRX* gene family during the evolution of *C. mollissima*, likely associated with adaptive evolution to particular environments (**Figures 1A, B**). Analysis of the physicochemical properties of the CmGRX proteins revealed significant variations (**Figure 1C**; **Supplementary Table 2**). The amino acid lengths of these proteins ranged from 97 aa (CmGrxC1) to 643 aa (CmROXY21), with molecular weights (MW) varying from 10.76 kDa to 73.53 kDa. The theoretical isoelectric points (pI) spanned from 4.83 (CmGrxS16c) to 9.47 (CmGrxC5), with 56.25% of the proteins (18 members) displaying pI values below seven, indicating their predominantly acidic nature. Most of the CmGRX proteins (23 members) exhibited an Instability Index above 40, suggesting they are likely to be unstable. The Aliphatic Index ranged from 62.96 (CmGrxS16h) to 111.32 (CmROXY17a), with 23 members exceeding 80, implying overall high thermal stability within the family. The GRAVY values varied from -0.769 (CmGrxS16k) to 0.308 (CmROXY17a), with 56.25% of the members being hydrophilic. Predictions of subcellular localization indicated that the majority of CmGRX members (23) are localized in the cytoplasm or plasma membrane, while others are distributed in the endoplasmic reticulum, mitochondria, and plastids, reflecting their diverse functional roles.

### Gene structure, conserved motif, and domain analysis

**Figure 2:**
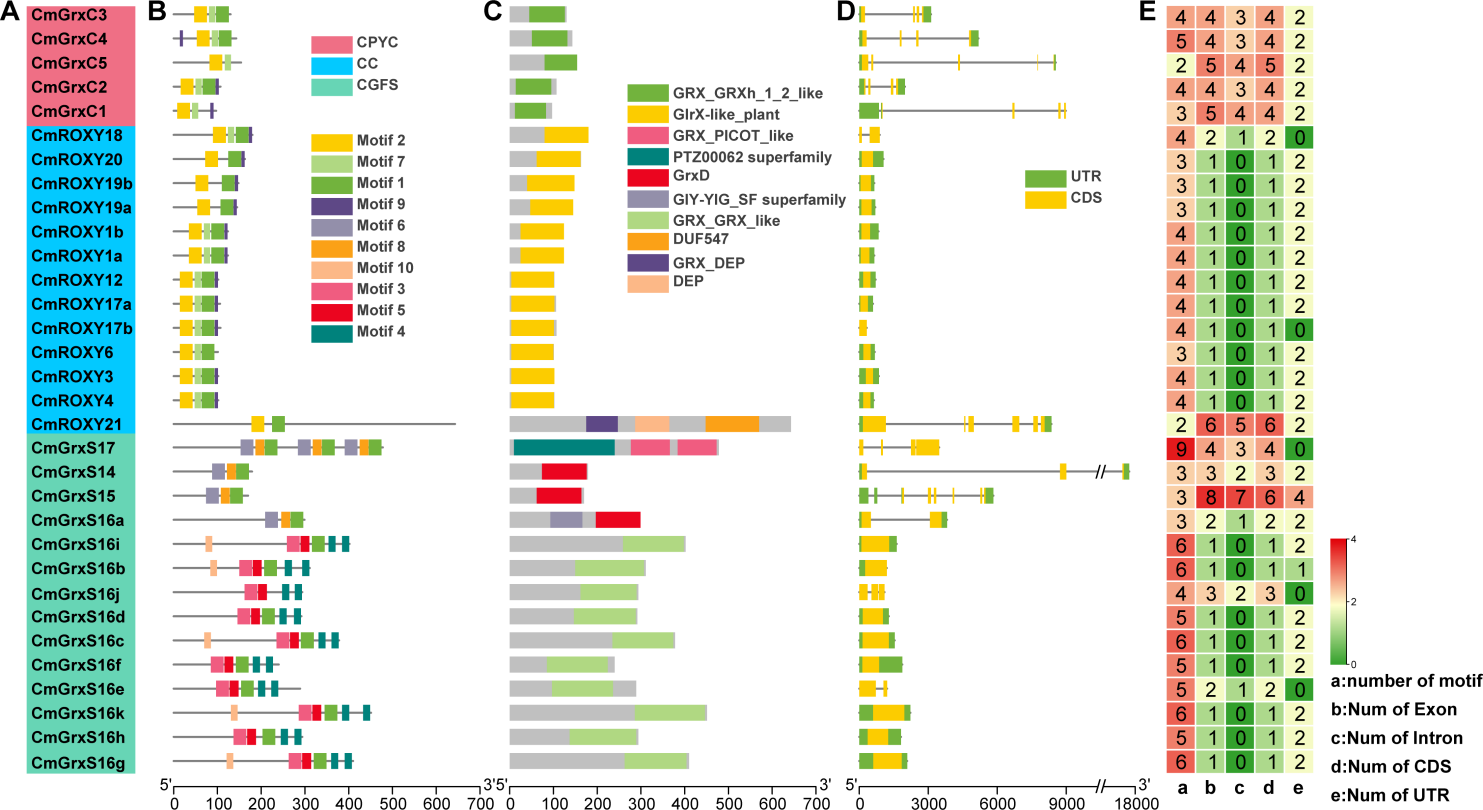
The conserved domains, gene structure, and conserved motifs distribution of *CmGRX* genes. **(A)** The subfamily of the *CmGRX* gene. **(B)** The conserved motifs of CmGRX proteins. **(C)** The conserved domains of *CmGRX* genes. **(D)** Gene structure of *CmGRX* genes. **(E)** The heatmap of motif number and gene structure for *CmGRX*.

To thoroughly analyze the structural characteristics and evolutionary conservation of the *CmGRX* gene family, we conducted a detailed investigation of conserved motifs, domains, and gene structures (exon-intron structure) (**Figure 2**). Our MEME analysis revealed 10 conserved motifs across the 32 CmGRX proteins, with individual members having between 2 and 9 motifs. Notably, the CGFS subfamily exhibited the highest structural complexity, containing an average of 5.14 motifs (**Figures 2A, B, E**). Domain analysis confirmed that all identified members possess the typical GRX domain (**Figure 2C**). An analysis of gene structures showed significant differences among the subfamilies, with the number of exons ranging from 1 to 8. The CC subfamily displayed a simplified exon-intron structure; with the exception of *CmROXY18* and *CmROXY21*, most members were characterized by single-exon genes. In contrast, the CPYC subfamily demonstrated more complex structures, containing 4 to 5 exons (**Figures 2D, E**). In summary, the subfamily-specific patterns in motif distribution, domain composition, and gene structure align closely with the phylogenetic classification, further supporting the functional conservation and specificity of each subfamily throughout evolution.

### Chromosomal location and duplication analysis

**Figure 3:**
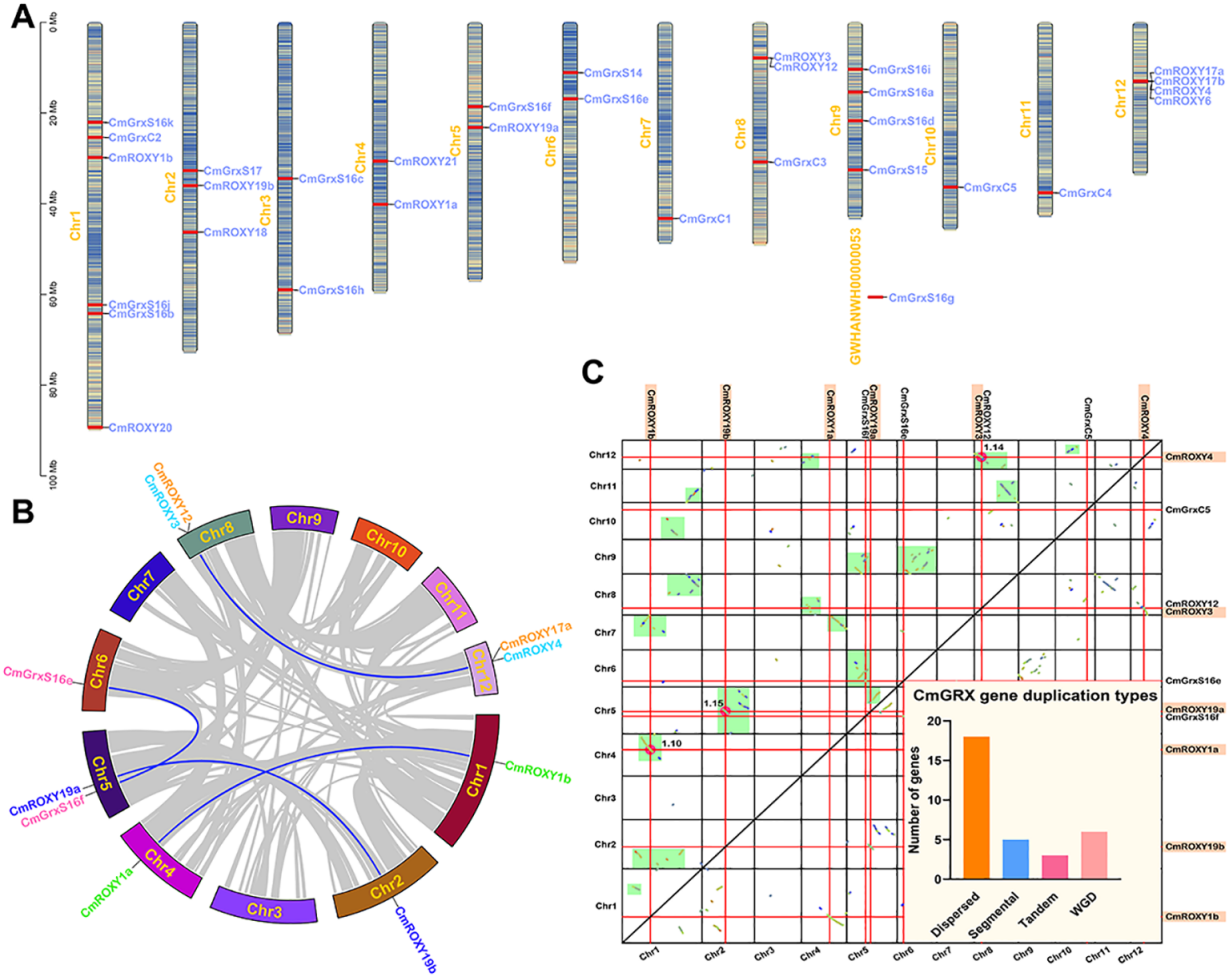
Chromosome distribution and duplication type analysis of *CmGRX* genes. **(A)** Chromosome distribution of *CmGRX* genes. The color of segments in the chromosomes shows the gene density of the corresponding region. **(B)** Collinear relationship within *C. mollissima* genome. **(C)** Homologous collinearity dot-plot within *C. mollissima* genome. The collinear blocks from WGD containing *CmGRX* genes are marked in the green boxes of the figure, and the block length and median Ks of the collinear blocks are marked. The genes highlighted in orange are identified as from WGD events. The bar chart in the bottom right corner of the dot-plot shows the number of *CmGRXs* for different duplication types.

Chromosomal mapping indicated that the 32 *CmGRX* genes are unevenly distributed across 12 chromosomes and one scaffold (GWHANWH00000053) (**Figure 3A**). Chromosome 1 housed the greatest number of members (6), followed by chromosomes 9 and 12 (4 each), and chromosomes 2 and 8 (3 each); chromosomes 7, 10, 11, and the scaffold each contained only one gene, while the remaining chromosomes each contained two. To explore the expansion mechanism of the *CmGRX* gene family, we performed intra-genomic collinearity analysis. This analysis identified 5 pairs of homologous *CmGRX* genes within the *C. mollissima* genome, highlighting the role of gene duplication in the family’s evolution (**Figure 3B**). To further evaluate the selective pressures acting on the duplicated *CmGRX* genes, Ka (non-synonymous substitution rate), Ks (synonymous substitution rate), and Ka/Ks ratios were calculated for the five collinear gene pairs (**Supplementary Table 3**). The Ka/Ks ratios ranged from 0.053 to 0.266, all substantially less than 1.0, indicating that these duplicated gene pairs have been subject to strong purifying selection, suggesting functional conservation after duplication events. Furthermore, by employing MCScanX in conjunction with the distribution of synonymous substitution sites (*Ks*) and collinear block analysis, using methods previously established (Yu et al., 2022; Liu et al., 2025a), we systematically classified the duplication types. The results indicated (**Figure 3C**) that dispersed duplication was the primary driving force behind the expansion of the *CmGRX* family, involving 18 genes (56.25%); this was followed by WGD and segmental duplication, involving 6 and 5 genes, respectively; only 3 genes originated from tandem duplication. This suggests that, unlike many stress-responsive gene families that rely on tandem duplication, the evolution of the *CmGRX* family in *C. mollissima* was more dependent on dispersed and segmental duplication events.

### Evolutionary analysis of the *CmGRX* gene family

**Figure 4:**
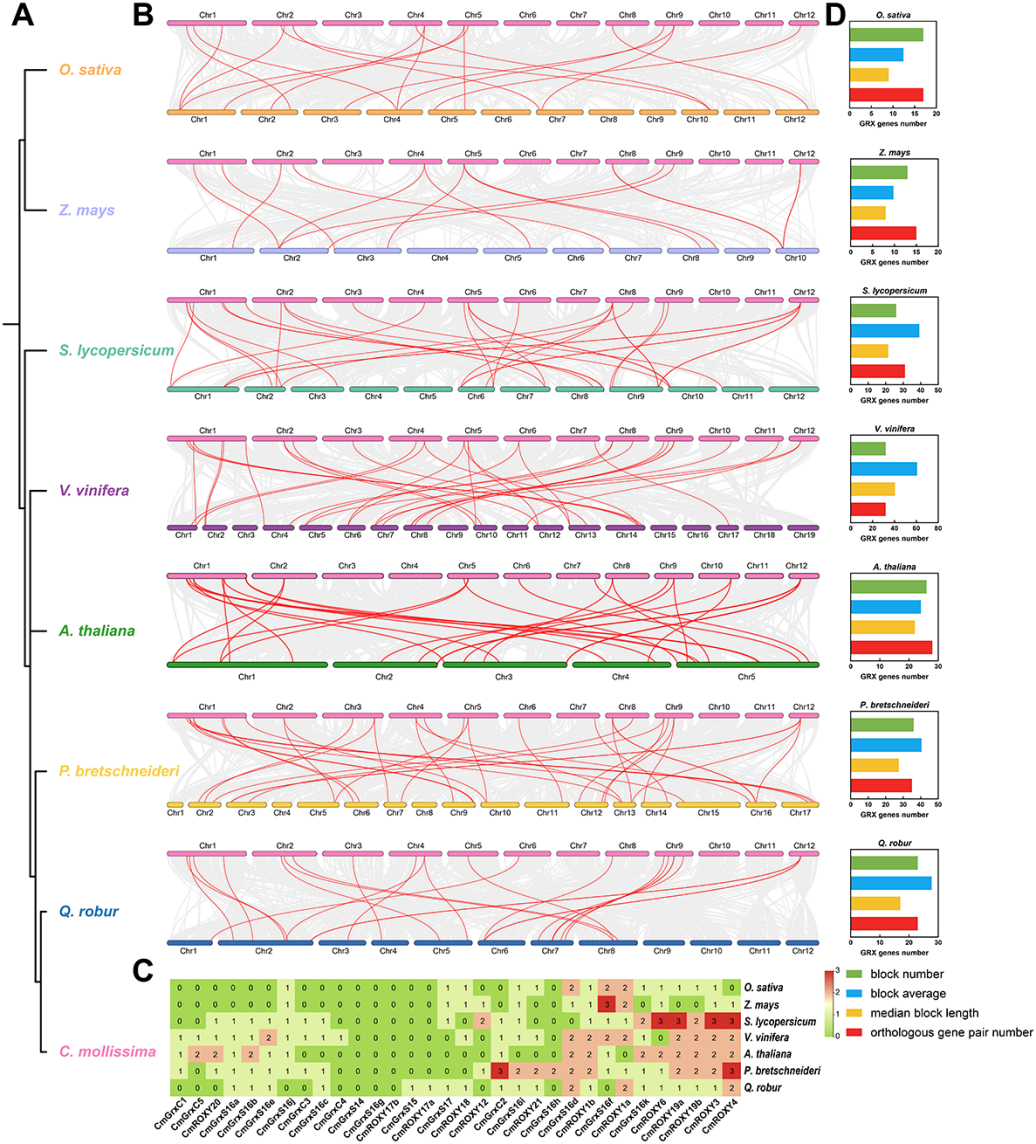
Collinear analyzes between the *CmGRX* genes and genes in seven representative plant species (*A. thaliana*, *O. sativa*, *Z. mays*, *Q. robur*, *V. vinifera*, *S. lycopersicum*, and *P. bretschneideri*). **(A)** The genetic relationship between eight plant species. **(B)** The dual collinear plot between *C. mollissima* and seven representative plant species. Gray lines in the background indicate collinear blocks within *C. mollissima* and other plant genomes, while red lines highlight collinear *GRX* gene pairs. Species names correspond to chromosome colors. **(C)** Heatmap of orthologous gene pairs between *C. mollissima* and seven other plant genomes. **(D)** The block number, median block length, average block length, and orthologous gene pair number between *C. mollissima* and other seven plant genomes.

To further explore the evolutionary origin and orthologous relationships of the *CmGRX* gene family, we constructed inter-genomic collinear maps between *C. mollissima* and two monocots (*O. sativa*, *Z. mays*) and five dicots (*S. lycopersicum*, *V. vinifera*, *A. thaliana*, *P. bretschneideri*, *Q. robur*) (**Figure 4A**). The analysis identified 17, 15, 31, 32, 28, 35, and 23 orthologous gene pairs between *CmGRX* and these seven species, respectively, involving 14, 12, 20, 23, 17, 23, and 20 *CmGRX* genes (**Figure 4B**; **Supplementary Tables 4**–**10**). Notably, some *CmGRX* genes exhibited a “one-to-many” collinear pattern in specific species, suggesting lineage-specific expansions. For example, *CmGrxS16f* has three collinear pairs in maize; *CmGrxC2* and *CmROXY4* each correspond to three homologs in pear; and in tomato, *CmROXY6*, *CmROXY19a*, *CmROXY3*, and *CmROXY4* each have three homologs. Conversely, *CmGrxS14*, *CmGrxS16g*, and *CmROXY17b* showed no collinear homologs in any tested species (**Figure 4C**). This absence of collinearity may result from neo-functionalization events in *C. mollissima*, rapid sequence divergence blurring homology, or specific gene loss in other lineages, implying their potential roles in *C. mollissima* adaptive traits (Tautz and Domazet-Lošo, 2011). In-depth analysis of syntenic blocks further revealed evolutionary affinities at the genomic level (**Figure 4D**; **Supplementary Table 11**–**S17**). *C. mollissima* shares 17 and 13 blocks with monocots, compared to 26, 32, 26, 36, and 23 blocks with the five dicots. Regarding block size, blocks shared with dicots were significantly larger than those with monocots; for instance, blocks shared with grape (*V. vinifera*) contained an average of 60.75 genes (median 40.5), whereas those with rice and maize contained only about 10–12 genes. As expected, *C. mollissima* demonstrated closer evolutionary conservation with dicots than with monocots in terms of orthologous gene numbers and syntenic block characteristics, consistent with its phylogenetic position.

### *Cis*-acting elements, TF regulatory network, and functional enrichment

**Figure 5:**
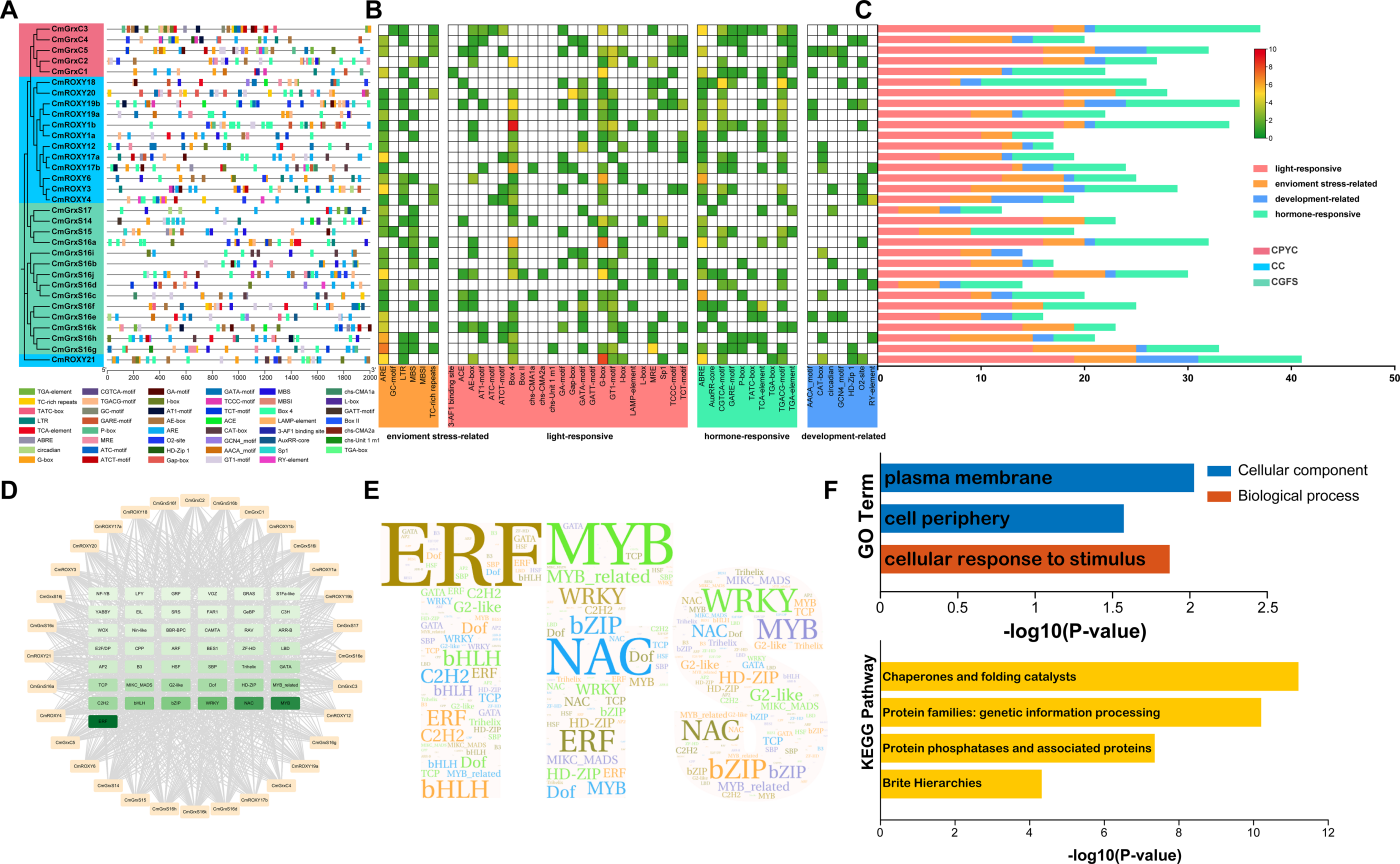
Prediction of *cis*-acting elements, TFs regulatory network, and GO/KEGG enrichment analysis in the promoters of *CmGRX* genes. **(A)** *Cis*-acting elements in the promoters of *CmGRX* genes. Various color symbols present different elements, and their position in the figure indicates their relative position on the promoter. **(B)** The relative proportions of different *cis* regulatory elements in the promoters of *CmGRX* genes are indicated in the chart. The same color represents *cis*-acting elements sharing identical or similar functions. **(C)** The number of various *cis*-acting elements in the promoters of each *CmGRX* genes. **(D)** The putative TFs regulatory network analysis of *CmGRX* genes. **(E)** Word cloud of predicted TFs interacting with *CmGRX* genes. The font size is positively correlated with the number of corresponding TFs. **(F)** GO and KEGG enrichment analysis of *CmGRX* genes.

To elucidate the upstream regulatory mechanisms influencing the *CmGRX* gene family, *cis*-acting elements were predicted within the 2000 bp promoter sequences of 32 members. In total, 47 types comprising 773 elements were identified and categorized into four groups: light response, hormone response, stress response, and growth and development (**Figures 5A, B**; **Supplementary Table 18**). Notably, several key elements were prevalent across the family members, including the anaerobic induction element ARE, the drought-inducible MYB binding site (MBS), core light-responsive elements Box 4 and G-box, and hormone-responsive elements ABRE (ABA) and CGTCA/TGACG-motif (MeJA). The widespread distribution of these elements suggests that the *CmGRX* family is finely regulated by multiple signals, including light, hormones, and environmental stresses. Furthermore, the abundance of elements varied significantly among the members (**Figure 5C**). For example, the promoters of *CmROXY21*, *CmGrxC3*, and *CmROXY19b* were enriched with numerous elements, indicative of complex and active expression patterns; in contrast, *CmGrxS16d*, *CmGrxS16i*, and *CmGrxS17* contained relatively fewer elements. Prediction of TF binding sites revealed an extensive and intricate transcriptional regulatory network involving 611 TFs from 43 families (**Figures 5D, E**; **Supplementary Table 19**). Network analysis identified ERF, MYB, and NAC family members as occupying central roles, consistent with their established functions in plant abiotic stress responses (Wang et al., 2021; Su et al., 2025; Xiong et al., 2025). Moreover, *CmGrxS16h*, *CmGrxS15*, *CmGrxS14*, and *CmROXY6* were predicted as key hub genes, regulated synergistically by a substantial number of TFs. Gene Ontology (GO) and Kyoto Encyclopedia of Genes and Genomes (KEGG) enrichment analyzes further validated the biological significance of these findings. GO analysis demonstrated significant enrichment of *CmGRX* genes in terms such as “cellular response to stimulus” and “plasma membrane,” supporting the discovery of abundant stress and hormone elements in their promoters. KEGG analysis classified these genes into “Chaperones and folding catalysts” and “Protein families: genetic information processing.” This classification not only supports the chaperone function of CmGRX in protein folding homeostasis and Fe-S cluster assembly but also suggests their involvement in complex genetic information processing and signal transduction networks through interactions with TFs (e.g., TGA) or protein phosphatases, thereby playing a central role in the synergistic regulation of multiple stress pathways (Ndamukong et al., 2007).

### Expression of *CmGRX* under stress and RT-qPCR validation

**Figure 6:**
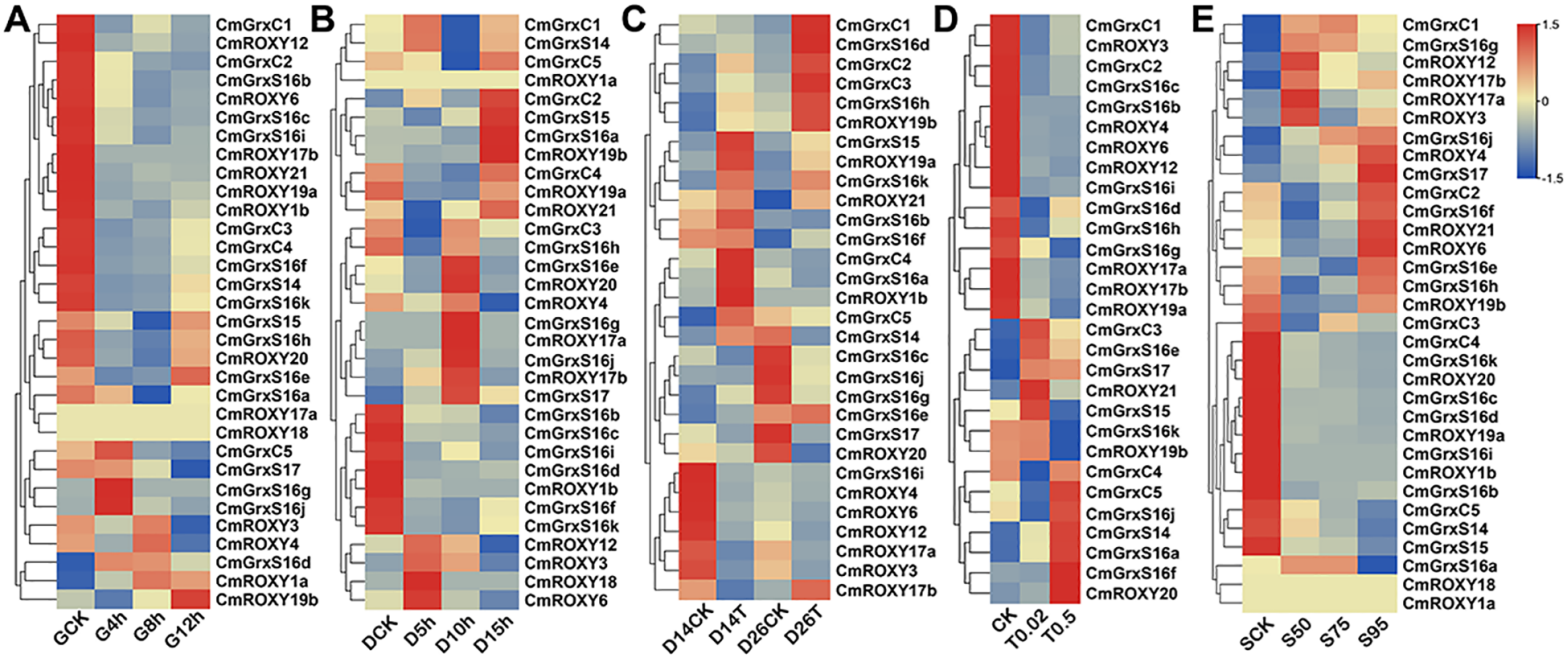
Heatmap of *CmGRX* gene expression under five abiotic stresses. **(A)** Heatmap of *CmGRX* gene expression under high temperatures. **(B)** *CmGRX* gene expression heatmap at low temperatures. **(C)** Heatmap of *CmGRX* gene expression under salt stress. **(D)** Heatmap of *CmGRX* gene expression under alkaline stress. **(E)** Heatmap of *CmGRX* gene expression under light-deprived conditions.

**Figure 7:**
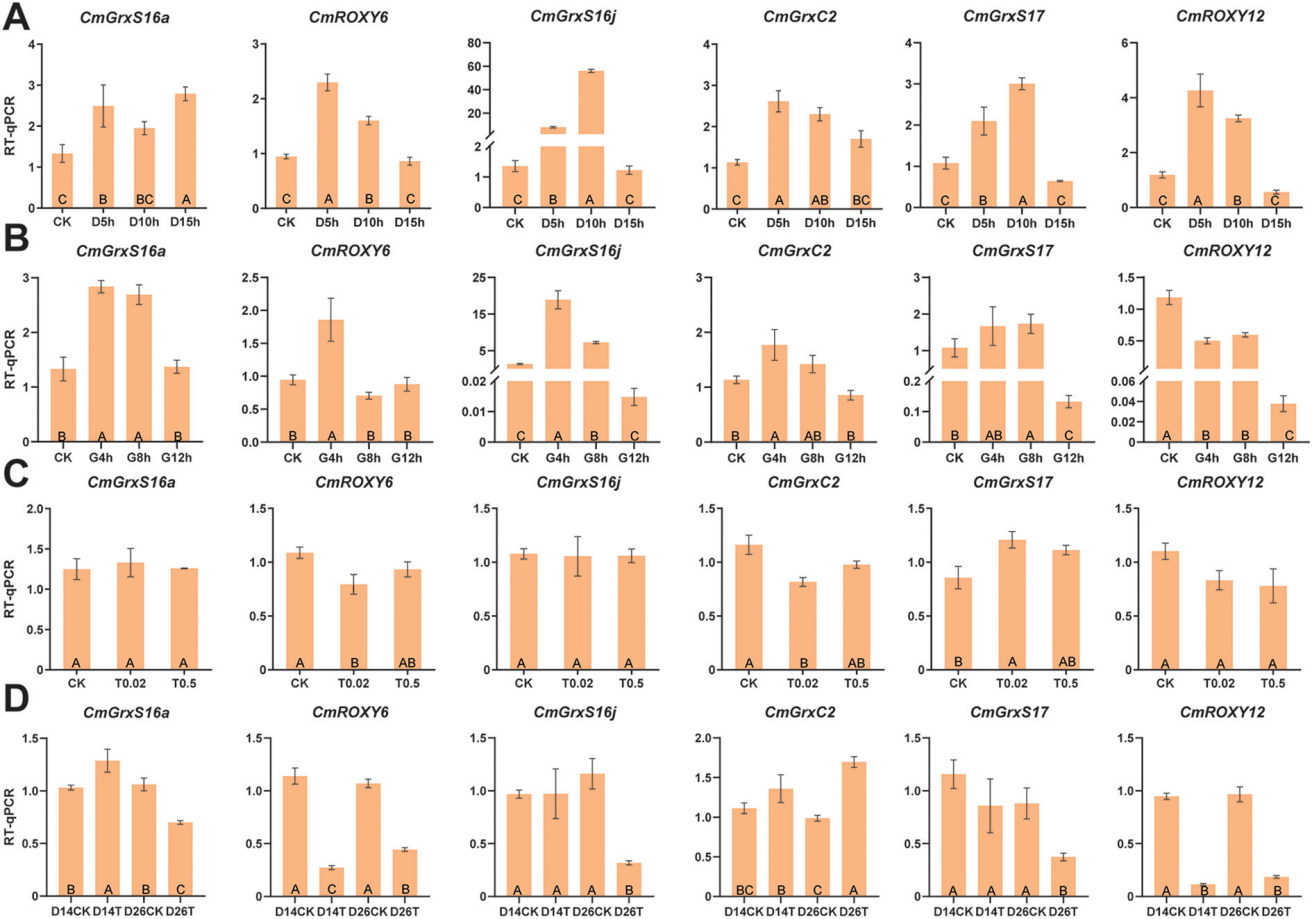
RT-qPCR validation of expression patterns for six *CmGRX* genes under four abiotic stresses. **(A)** Expression profiles under low temperature stress. **(B)** Expression profiles under high temperature stress. **(C)** Expression profiles under alkaline stress. **(D)** Expression profiles under salt stress. Orange bars linked to the left y-axis show relative expression levels determined by RT-qPCR. Different uppercase letters denote statistically significant differences at *P* < 0.05 for RT-qPCR results.

To elucidate the dynamic response patterns of the *CmGRX* family under complex environments, we analyzed their expression profiles in response to high temperature, low temperature, salt, alkaline, and shading stresses using transcriptome data from the N11–1 reference genome (**Figure 6**). The results demonstrated that members of the *CmGRX* family exhibited significant and diverse responses to stress. Under high-temperature conditions, the transcription of most genes was suppressed; notably, *CmGrxC2*, *CmGrxS16a*, *CmGrxS17*, *CmROXY12*, and *CmROXY6* were variably downregulated, with *CmROXY12* and *CmROXY6* showing the most substantial reductions in expression (FPKM values decreased from 24.61 and 32.95 to 0.15 and 7.39, respectively). Conversely, low-temperature stress triggered notable expression fluctuations; for instance, *CmGrxS16j* exhibited a surge-then-decline pulse response (peaking at 517.83), while *CmGrxC2*, *CmGrxS16a*, and *CmGrxS17* demonstrated an upregulation trend, indicating their pivotal roles in cold adaptation. Under salt and alkaline stresses, *CmROXY12* and *CmROXY6* showed significant downregulation patterns similar to those observed under high-temperature stress, suggesting that these genes may respond to multiple stresses through common signaling pathways. Furthermore, shading stress markedly induced the expression of *CmGrxS16j* and *CmGrxS17*, which increased progressively with the intensity of shading, indicating their potential roles in metabolic regulation under low-light conditions. To validate the reliability and accuracy of the RNA-seq data, six *CmGRX* genes representing all three subfamilies were selected for RT-qPCR validation based on their significant differential expression across multiple stress conditions in RNA-seq data (**Figure 7**). The comparative results showed that the relative expression trends detected by RT-qPCR were highly consistent with the RNA-seq FPKM values, thereby confirming the reliability of the transcriptomic data in this study.

## Discussion

The GRX system, comprising GRX, GSH, glutathione reductase (GR), and NADPH, forms a crucial redox regulatory module (Rouhier et al., 2008; Wu et al., 2017b). Originally identified as electron donors for ribonucleotide reductase in *Escherichia coli*, GRXs are now recognized as a superfamily of functionally diverse GSH-dependent oxidoreductases that play broad roles in redox signaling and Fe-S cluster assembly across various organisms (Holmgren, 1976; Ogata et al., 2021). Although the genome-wide identification and functional characterization of GRX families have been accomplished in several plant species (Garg et al., 2010; Malik et al., 2020; Li et al., 2021; Guo et al., 2025; Liu et al., 2025b), the composition, evolutionary history, and biological functions of the GRX family in *C. mollissima* remain unexplored. Consequently, this study represents the first comprehensive genome-wide analysis of the *CmGRX* family, aimed at uncovering its evolutionary characteristics and investigating its potential roles in abiotic stress responses, thereby establishing a theoretical basis for further functional research.

We identified 32 *CmGRX* genes within the genome of *C. mollissima* and categorized them into three subfamilies based on phylogenetic analysis (**Figure 1**; **Supplementary Table 2**). Comparative analysis across species revealed notable lineage-specific expansion patterns: the CGFS subfamily in *C. mollissima* has undergone significant expansion compared to *A. thaliana*, whereas the plant-specific CC subfamily has experienced a contraction. Such remodeling of the gene family structure likely reflects the evolutionary adaptations of *C. mollissima* to particular metabolic requirements or environmental stressors. Physicochemical and structural analyzes indicated that, despite substantial variations in gene length, members within each subfamily maintain high conservation in terms of gene structure, motifs, and domain composition (**Figure 2**). Notably, members of the CC subfamily exhibited a highly streamlined exon-intron structure, with an average of only 1.46 exons per gene. According to the Genome Design Hypothesis, such compact gene structures are typically favored in genes that need to respond swiftly to environmental stimuli, thereby enhancing transcriptional elongation rates and splicing efficiency (Jeffares et al., 2008). Considering that abiotic stresses often trigger transient and intense bursts of ROS, plants must quickly activate molecular defense mechanisms (Apel and Hirt, 2004; Foyer and Noctor, 2005; Gill and Tuteja, 2010; Miller et al., 2010; Mittler et al., 2022). We therefore propose that the streamlined structural configuration of *CmGRX* genes facilitates their rapid activation upon detection of stress signals. This would enable the swift accumulation of CmGRX proteins to activate the antioxidant network and mitigate oxidative damage. In addition to their structural benefits, *CmGRX* genes are intricately regulated by complex upstream transcriptional networks (**Figure 5**). The precise binding of TFs to promoter elements constitutes a fundamental mechanism of gene regulation (Riechmann et al., 2000; Franco-Zorrilla et al., 2014). Our predictions for the transcriptional network are highly consistent with the results from *cis*-element analysis, demonstrating that ERF, MYB, and NAC family members play central roles in the regulation of *CmGRX*, as evidenced by the enrichment of MBS and ethylene-responsive elements in the promoter regions. It is established that MYB and NAC TFs are crucial in plant responses to abiotic stress, facilitating transcriptional reprogramming in response to drought, high salinity, and extreme temperatures (Abe et al., 2003; Olsen et al., 2005), while ERF factors are essential mediators of ethylene signaling in stress defense (Müller and Munné-Bosch, 2015). This coordinated regulation by multiple TF families indicates that the *CmGRX* family is not controlled by a single pathway. Instead, it appears to function as a crucial downstream node that integrates signals from multiple upstream stress signaling pathways, including those mediated by ERF, MYB, and NAC. This regulatory network ensures precise spatiotemporal control of *CmGRX* expression in *C. mollissima*, maintaining a fine cellular redox balance amidst complex environmental fluctuations. Integrative analysis further revealed a correspondence between promoter *cis*-elements and expression patterns. For example, *CmGrxS16a*, harboring LTR, ABRE, and MBS elements, was significantly upregulated under cold stress. *CmGrxS16j*, enriched with ARE and ABRE elements, was markedly induced under shading. *CmROXY6* and *CmROXY12*, both containing ABRE, ARE, and MBS elements, showed parallel downregulation under high-temperature, salt, and alkaline stresses, suggesting co-regulation through shared ABA- and MYB-mediated pathways. These findings provide a mechanistic link between the upstream regulatory architecture and the stress-responsive expression of *CmGRX* genes.

The identification of 32 *CmGRX* genes reveals a family size similar to that of *A. thaliana* (31) (Rouhier et al., 2004) and *C. annuum* (35) (Guo et al., 2025), but significantly smaller than in *O. sativa* (48) (Garg et al., 2010), *G. hirsutum* (127) (Malik et al., 2020), and *M. acuminata* (64) (Li et al., 2021). This difference indicates significant variation in the expansion of GRX families across different plant lineages. Gene duplication is a core mechanism driving gene family expansion and functional divergence (Ren et al., 2018; Qiao et al., 2019; Schilling et al., 2020; Chen et al., 2023; Shan et al., 2025). Collinearity analysis identified five pairs of homologous *CmGRX* genes. Notably, 56.25% of *CmGRX* genes originated from dispersed duplication, indicating that dispersed duplication, rather than whole-genome duplication (WGD) or tandem duplication, is the dominant force driving the expansion of the *CmGRX* family (**Figure 3**). Interspecific collinearity analysis further revealed the evolutionary characteristics of *CmGRX* (**Figure 4**). As expected, the collinearity level between *C. mollissima* and dicots was significantly higher than with monocots, consistent with angiosperm phylogeny and corroborating early dicot divergence events (Jaillon et al., 2007). However, micro-level statistics of syntenic blocks showed that the number of orthologous pairs does not strictly follow phylogenetic distance. This nonlinear relationship may be attributed to lineage-specific genome fractionation rates and chromosomal rearrangement events, as reported in other *C. mollissima* gene families (Freeling, 2009; Liu et al., 2025a; Yu et al., 2025a, 2025b). Specifically, the evolutionary retention pattern shows polarization: some genes, such as *CmGrxS16f*, *CmGrxC2*, and *CmROXY6*, retained multiple orthologous copies in single species, suggesting they may perform dosage-sensitive core housekeeping functions; conversely, genes like *CmGrxS14*, *CmGrxS16g*, and *CmROXY17b* showed no collinear homologs in any tested species (**Figure 4C**). These *C. mollissima*-specific or rapidly diverging genes may have originated from post-speciation adaptive evolution to meet specific ecological niche requirements. Furthermore, Ka/Ks analysis of the five collinear *CmGRX* gene pairs revealed that all pairs have undergone strong purifying selection (Ka/Ks = 0.053–0.266), indicating significant functional constraints on these duplicated genes throughout evolution. This is consistent with the essential role of GRXs in maintaining cellular redox homeostasis, where deleterious mutations that disrupt protein function would be effectively eliminated by natural selection (Hurst, 2002).

Transcriptomic analysis revealed marked differences in the expression of *CmGRX* under various abiotic stresses, notably with *CmROXY12*, *CmROXY6*, *CmGrxS16j*, *CmGrxC2*, *CmGrxS16a*, and *CmGrxS17*, which exhibited substantial transcriptional variations (**Figure 6**). Based on sequence homology and similarities in expression patterns, functional conservation can be inferred. Fe-S clusters are well-recognized as electron carriers and cofactors for numerous enzymes, playing critical roles in regulating oxidative stress, genome stability, and cellular development (Balk and Pilon, 2011; Balk and Schaedler, 2014). In *A. thaliana*, CGFS-type GRX proteins have been demonstrated to confer stress tolerance by participating in the biosynthesis and homeostasis of Fe-S clusters (Cheng et al., 2006; Bandyopadhyay et al., 2008; Iñigo et al., 2016). For instance, transcription of *AtGrxS17* is significantly induced by high temperatures, and its loss-of-function mutant is hypersensitive to heat stress, whereas heterologous expression of *AtGrxS17* significantly enhances heat tolerance and ROS scavenging in tomatoes (Wu et al., 2012; Hu et al., 2015; Wu et al., 2017b). In this study, the consistent upregulation of *CmGrxS17* under temperature stress strongly suggests a similar function in the heat tolerance mechanisms of *C. mollissima*. Additionally, members of the CGFS subfamily, *CmGrxS16a* and *CmGrxS16j*, displayed significant expression changes under both temperature and shading stresses. Current research suggests that plant-specific CC-type glutaredoxins (ROXYs) primarily function as transcriptional co-regulators, recruiting TGA TFs to refine gene expression networks and playing pivotal roles in the morphogenesis of floral organs and in response to biotic and abiotic stresses (Gutsche et al., 2015). For example, *AtROXY1* and *AtROXY2* are established as key regulators of anther development in *A. thaliana* (Xing and Zachgo, 2008), while *AtROXY19* (*GRX480*) and *AtROXY18* (*GRXS13*) show broad stress-inducible properties, responding to a variety of biotic and abiotic stimuli (Ndamukong et al., 2007; La Camera et al., 2011; Huang et al., 2016). Given the central status of CC-type GRXs in model plants, our findings that *CmROXY12* and *CmROXY6* cluster closely with *A. thaliana* CC-type members and exhibit significant transcriptional alterations under high temperature and saline-alkali stresses underscore the importance of their role. This conserved pattern of expression strongly implies that *CmROXY12* and *CmROXY6* may integrate environmental signals to regulate stress tolerance in *C. mollissima* through conserved TGA-mediated pathways. Conversely, for the dithiol (CPYC-type) members, *AtGrxC2* has been shown to catalyze the S-glutathionylation of the receptor kinase BAK1 *in vitro*, thus inhibiting its kinase activity (Bender et al., 2015). In this study, *CmGrxC2* demonstrated a significant downregulation under low-temperature stress, suggesting an important role in redox reactions under such conditions. In conclusion, this research not only bridges gaps in the genomic knowledge of the *C. mollissima* GRX family but also highlights its unique evolutionary traits and expression patterns, which are regulated by multiple stress-responsive TFs. These insights provide crucial gene targets and theoretical support for enhancing abiotic stress tolerance in *C. mollissima* through precision molecular breeding.

## Data Availability

The RNA-seq data presented in the study are deposited in the NCBI BioProject and GSA repositories. The accession numbers are as follows: temperature stress (PRJNA1166987), salt stress (PRJNA1363574), alkaline stress (PRJNA1384844), and shading treatment (GSA: CRA022911).

## Supplementary material

Supplementary Table 1 Primers used for qPCR analysis.

Supplementary Table 2 Accession numbers and characteristics of 32 *CmGRX* genes in *Castanea mollissima*.

Supplementary Table 3 The duplication model and calculation of Ka and Ks ratios of duplicated *CmGRX* gene pairs.

Supplementary Table 4 The orthologous gene pairs containing *CmGRX* in the cross of *C. mollissima* and *V. vinifera*.

Supplementary Table 5 The orthologous gene pairs containing *CmGRX* in the cross of *C. mollissima* and *A. thaliana*.

Supplementary Table 6 The orthologous gene pairs containing *CmGRX* in the cross of *C. mollissima* and *Q. robur*.

Supplementary Table 7 The orthologous gene pairs containing *CmGRX* in the cross of *C. mollissima* and *O. sativa*.

Supplementary Table 8 The orthologous gene pairs containing *CmGRX* in the cross of *C. mollissima* and *S. lycopersicum*.

Supplementary Table 9 The orthologous gene pairs containing *CmGRX* in the cross of *C. mollissima* and *P. bretschneideri*.

Supplementary Table 10 The orthologous gene pairs containing *CmGRX* in the cross of *C. mollissima* and *Z. mays*.

Supplementary Table 11 The collinear blocks containing *CmGRX* identified between *C. mollissima* and *V. vinifera*.

Supplementary Table 12 The collinear blocks containing *CmGRX* identified between *C. mollissima* and *A. thaliana*.

Supplementary Table 13 The collinear blocks containing *CmGRX* identified between *C. mollissima* and *Q. robur*.

Supplementary Table 14 The collinear blocks containing *CmGRX* identified between *C. mollissima* and *O. sativa*.

Supplementary Table 15 The collinear blocks containing *CmGRX* identified between *C. mollissima* and *S. lycopersicum*.

Supplementary Table 16 The collinear blocks containing *CmGRX* identified between *C. mollissima* and *P. bretschneideri*.

Supplementary Table 17 The collinear blocks containing *CmGRX* identified between *C. mollissima* and *Z. mays*.

Supplementary Table 18 Information of *cis*-acting elements predicted from 2000 bp upstream regions of *CmGRX* genes.

Supplementary Table 19 Predictive transcription factor (TFs) analysis of *CmGRX* genes.
